# GnRH agonist triggering affects the kinetics of embryo development: a comparative study

**DOI:** 10.1186/s13048-016-0229-8

**Published:** 2016-04-08

**Authors:** Ali Sami Gurbuz, Funda Gode, Mehmet Sukru Uzman, Betul Ince, Melek Kaya, Necati Ozcimen, Emel Ebru Ozcimen, Ali Acar

**Affiliations:** Novafertil IVF Centre, Yeni Meram yolu No:75, Meram, Konya Turkey; Department of Obstetrics and Gynecology, Izmir University Hospital, Izmir, Turkey; Department of Obstetrics and Gynecology, Baskent University Hospital, Konya, Turkey; Department of Obstetrics and Gynecology, Necmettin Erbakan University, Konya, Turkey

**Keywords:** Agonist trigger, Oocyte, Embryo quality, Time lapse, Morphokinetic

## Abstract

**Background:**

To evaluate the effects of an ovulation triggering agent, human chorionic gonadotropin (hCG), versus a gonadotropin-releasing hormone agonist (GnRHa) on early embryo development *in vitro* using a time-lapse system.

**Methods:**

Retrospective analysis of a prospectively collected database. A total of 739 embryos from 152 infertile couples undergoing intracytoplasmic sperm injection cycles.

Interventions : Embryo culture in a time-lapse incubator (EmbryoScope, Vitrolife, Göteborg, Sweden). Main Outcome Measures: Embryo morphokinetic parameters.

**Results:**

In the 152 women, 252 embryos were derived from GnRHa-triggered cycles compared with 487 embryos derived from hCG-triggered cycles. Time-lapse analysis revealed that embryos from cycles triggered by a GnRHa cleaved faster than embryos derived from hCG-triggered cycles.

**Conclusion:**

Triggering with a GnRHa in *in vitro* fertilization cycles affects embryo kinetics.

## Background

Embryo quality is one of the most important factors affecting the success of *in vitro* fertilization (IVF). Currently, embryo quality is determined using morphological evaluation methods, and in most circumstances the embryologist’s decision is the last step in choosing the embryo that is transferred to the patient. Although morphological evaluation has been the gold standard for many years, it is a subjective process with inter- and intra-observer variability [1]. It is also a static evaluation method, and some abnormalities cannot be detected over the time interval involved in embryo evaluation. Time-lapse monitoring is a new technology that enables dynamic, more objective evaluation of embryos [2, 3].

The treatment protocol and duration and the type and dosage of drugs are clinician-dependent factors that might affect oocyte and embryo quality. Initially, IVF treatment was performed in a natural cycle; however, over the last 20 years many different treatment protocols have been used [4]. Gonadotropin-releasing hormone agonists (GnRHa) have long been used to inhibit premature luteinizing hormone (LH) release. In the last decade, however, a GnRH antagonist protocol has become preferred for pituitary desensitization worldwide, because it is a more patient friendly approach that also reduces the risk of ovarian hyperstimulation syndrome (OHSS) [5]. Another advantage of antagonist cycles is they enable the use of a GnRHa for triggering final oocyte maturation. There are some physiological differences between human chorionic gonadotropin (hCG) and GnRHa triggers. Unlike hCG triggering of final oocyte maturation, GnRHa triggering is a more physiological approach, eliciting a surge of gonadotropins similar to that of the natural mid-cycle surge [6]. The serum LH and follicle-stimulating hormone (FSH) levels rise after 4 and 12 h, respectively, and are elevated for 24–36 h. The amplitudes of the surge are similar to those observed during the normal menstrual cycle [7]. However, hCG-mediated LH activity persists for several days into the luteal phase [8, 9].

Consequently, the two triggering agents affect oocyte maturation in different ways. Does this difference affect oocyte development and subsequent embryo quality? A recent study showed that GnRHa triggering results in the retrieval of more metaphase II (MII) oocytes compared with hCG triggering [8]. This was related to the endogenous FSH surge elicited along with the LH surge after GnRHa triggering [8, 9]. Recently, Munoz et al. explored the effect of controlled ovarian stimulation and the ovulation triggering factor (GnRHa + hCG triggering versus GnRH antagonist + GnRHa triggering) on embryo development and kinetics [10]. They reported that embryos from cycles involving GnRH antagonist + GnRHa treatment cleaved faster than embryos derived from patients co-treated with a GnRHa + hCG. Their findings might be related to either the stimulation protocol or the triggering agent. Insufficient data have compared the steps following GnRHa- and hCG-triggered cycles using the same stimulation protocol, including fertilization and embryo developmental kinetics.

Therefore, this study compared the effects of hCG and GnRHa triggering on embryo developmental kinetics in antagonist cycles.

## Methods

This retrospective cohort study analyzed the data on embryos from 152 couples undergoing intracytoplasmic sperm injection cycles from May 2014 to May 2015. The study was conducted at the Novafertil IVF Center in Konya, Turkey. The study protocol was approved by the Institutional Review Board. Exclusion criteria were endometriosis, poor ovarian reserve, azospermia, age > 36 years.

### Ovarian stimulation

All patients followed a GnRH antagonist protocol. Ovarian stimulation was initiated with recombinant FSH (Puregon; MSD, Turkey or Gonal-F; Merck Serono, Turkey) on day 2 or 3 of the cycle and continued until the day of ovulation trigger. Cycles were monitored using ultrasound scanning. A GnRH antagonist, either ganirelix (Orgalutran; MSD, Turkey) or cetrorelix (Cetrotide; Merck Serono, Turkey), was administered when the leading follicle attained a maximum diameter of 14 mm. When at least two follicles had reached diameters of 17 mm, final oocyte maturation was triggered by administering 0.2 mg of the GnRHa triptorelin (Gonapeptyl; Ferring, Turkey) in Group 1 or recombinant hCG (Ovitrelle; Merck Serono, Turkey) in Group 2.

### Oocyte retrieval and intracytoplasmic sperm injection

Transvaginal oocyte retrieval was performed 35 h after triggering. Intracytoplasmic sperm injection was performed in all patients. Embryos were evaluated on third day, and up to two embryos were transferred per patient on day 3 of development. All embryos were selected according to their morphological evaluation and embryo kinetics data were not used for embryo selection. For luteal support, all patients in Group 1 were given 90 mg progesteron gel (8 %) (Crinone gel, Merck Serono, Turkey), 50 mg/day of intramuscular progesterone (Progestan amp 50 mg, Koçak Farma, Turkey) and 4 mg/day estradiol hemihydrate (Estrofem 2 mg, Novo Nordis; Turkey). All patients in Group 2 were given 90 mg progesteron gel (8 %) (Crinone gel, Merck Serono, Turkey).

### Time-lapse imaging

Images of each embryo were acquired every 20 min in seven focal planes, initiated after insemination. The images were analyzed using Embryo Viewer software, which annotates embryonic developmental events with the corresponding time in hours after microinjection. The times from insemination to the following events were analyzed: when two pronuclei were visible (2PN); when second polar body was detected (PB2), pronuclear fading (PNF), when both pronuclei disappear; first cleavage, when the zygote divides into two cells (t_2_); and when cleavage giving rise to 3 to 9 cells is observed for the first time (t_3_ to t_9_, respectively). The intervals between two consecutive cleavages were also analyzed. The duration of the second cell cycle (cc_2_ = t_3_ – t_2_) is the time from the division into a two-blastomere embryo until the time to the division into a three-blastomere embryo, and second synchrony (s_2_ = t_4_ – t_3_) is the time from this division into a four-blastomere embryo.

### Morphokinetic categories

Recently, Meseguer et al. reported the optimal ranges of the morphokinetic parameters t_5_, s_2_, and cc_2_ [11]. The ranges of these parameters used in this current study were as follows: t_5_ = 48.8–56.6 h, s_2_ < 0.76 h, and cc_2_ < 12 h. Embryos within these ranges were described as optimal embryos having the highest probability of implantation.

### Statistical analysis

The Statistical Package for the Social Sciences 20 (SPSS, Chicago, USA) was used for the statistical analysis. The results were analyzed using Student’s *t-*test to compare timings and the chi-square test to compare proportions. A *p*-value of less than 0.05 was considered to be statistically significant.

## Results

A total of 152 women were included in this study. The groups did not differ in terms of age, body mass index (BMI), day 3 FSH, total FSH dose, peak E2 levels, numbers of oocytes, MII oocytes, embryos, embryos transferred, stimulation days, or implantation and pregnancy rates (Table 1).

**Table 1:** Demographic characteristics of the two study groups

	GnRHa triggering (*n* = 50)	hCG triggering (*n* = 102)	*p*
Age	28.35 (4.03)	28.70 (2.33)	NS
BMI	24.3 (4.08)	23.7 (3.82)	NS
Day3 FSH	6.1 (2.8)	5.2 (2.1)	NS
Total FSH dose	1895 (634)	2091 (642)	NS
Peak E2 levels	3461 (1722)	3344 (1648)	NS
No. oocytes	16.78 (5.27)	15.54 (6.45)	NS
No. oocytes MII	11.03 (5.28)	10.74 (4.26)	NS
No. embryos	7.16 (3.61)	7.27 (331)	NS
No. embryos transferred	1.74 (0.25)	1.63 (0.25)	NS
Stimulation days	9.74 (2.16)	10.30 (1.45)	NS
Pregnancy rate (%)	64 (32/50)	63.7 (65/102)	NS
Clinical pregnancy rate (%)	50 (25/50)	45 (46/102)	NS
Implantation rate (%)	40 (35/86)	35 (58/165)	NS

Results are presented as means (SD) when appropriate

*NS* no statistically significant differences were found, *BMI* body mass index, *FSH* follicle stimulating hormone, *E2* Estradiol

When the embryo developmental kinetics were evaluated (Table 2), some early developmental events occurred significantly later in embryos derived from cycles triggered with hCG (*n* = 102 patients, *n* = 487 embryos) than in embryos derived from cycles triggered with a GnRHa (*n* = 50 patients, *n* = 252 embryos). The times from insemination to tPB2, tPNF, t_2_, t_3_, t_5_, and t6 were significantly shorter in the GnRHa triggered group (Group 1). The groups did not differ significantly in terms of t_4_, t_7_, t_8_, t_9_, cc_2_, and s_2_, but cc_3_ was significantly shorter in Group 1 than in Group 2.

**Table 2:** Embryo developmental kinetics according to the type of oocyte maturation triggering agent

	GnRHa triggering (Group 1 n:252)	hCG triggering (Group 2 n:487)	*P*
t_PB2_	4.5 (1.7)	5.4 (1.8)	0.000
t_PNf_	24.8 (5.2)	26.7 (5.2)	0.000
t_2_ (h)	32.2 (6.2)	34.3 (5.5)	0.009
t_3_ (h)	38.5 (6.0)	39.9 (5.1)	0.027
t_4_ (h)	40.6 (6.3)	41.7 (5.3)	0.101
t_5_ (h)	47.0 (7.9)	50.8 (7.1)	0.000
t_6_ (h)	52.1 (7.3)	54.6 (6.2)	0.002
t_7_ (h)	57.5 (8.2)	57.8 (5.2)	0.871
t_8_ (h)	63.2 (6.3)	62.5 (5.1)	0.589
t_9+_ (h)	64.5 (5.9)	64.9 (4.8)	0.996
cc_2_ (h)	6.9 (5.9)	6.3 (4.1)	0.321
s_2_ (h)	2.6 (4.2)	2.0 (2.2)	0.151
cc_3_ (h)	9.7 (6.1)	11.5 (4.5)	0.006
t_4_ – t_2_ (h)	9.5 (6.4)	8.6 (4.6)	0.071
t_8_ – t_4_ (h)	24.3 (6.3)	23.5 (5.1)	0.251

Results are presented as means(SD) when appropriate

*t* time, *h* hour, *t*_*PB2*_ appearance of second polar body *t*_*PNf*_ both pronuclei faded, *cc* cell cycle *s*, synchrony

The percentage of optimal embryos based on the relevant morphokinetic variables were compared according to type of triggering agent. Variables analyzed were the time of cleavage to five cells (t_5_), second cell cycle (cc_2_), duration of the period as a two-cell blastomere embryo (t_3_ – t_2_), and synchrony in division from two-cell blastomere embryos to four-cell blastomere embryos (s_2_). There were no significant difference between groups according to t5. However percentage of optimal embryos according to s2 and cc2 were significantly higher in GnRH agonist group (Group1) than hCG triggering group (*p* < 0.05) (Table 3)

**Table 3:** Percentages of optimal embryos whose cleavages are included in optimal timing ranges with a predicted higher implantation potential (Meseguer et al. 2011) according to type of triggering

Embryo category	GnRHa triggering	hCG triggering	*p*
T5 (%)	15.4	17.4	NS
S2 (%)	42.0	24.8	0.000
CC2 (%)	52.3	43.1	0.005

Data are presented as % (*n*) for each category. The proportions of optimal embryos in each category were compared using the *χ* ^2^ test. NS. no statistically significant differences were found

## Discussion

Embryo competence depends on oocyte quality, which is affected by different factors, including the treatment modality [12, 13]. We do not know the exact effect of the dosages and types of drugs on oocytes in IVF. Therefore, it was suggested that mild stimulation protocols and natural cycles would reduce aneuploidy rates and increase embryo quality [12]. However, every step during ovulation induction could affect the oocytes and subsequent embryo development.

Triggering oocyte maturation is the last important step of ovulation induction. For a long time, hCG has been used as a triggering agent because of its homology with LH and extended half-life. Although the extended half-life of this molecule might be advantageous for luteal support, its effect on oocyte maturation is not clear and hCG-mediated LH activity differs from natural cycles. Recently, GnRH agonists have been used as the triggering agent, especially in patients at a high risk of OHSS in GnRH antagonist cycles. The GnRHa displaces the GnRH antagonist from the GnRH receptor, inducing initial activation (flare-up) of LH and FSH, similar to that of the natural cycle before receptor downregulation [14]. This seems to be a more physiological mode of oocyte maturation. We examined whether there is a difference in the development of these oocytes after GnRHa triggering. Limited data are available on this subject in the literature. To our knowledge, this is the first study to evaluate embryo kinetics after the use of different oocyte-triggering agents in patients undergoing the same treatment protocol. Our results confirmed that the oocyte-triggering agent affected the early developmental kinetics of oocytes and embryos.

Interestingly, we found that the time intervals during early embryo development were shorter in GnRHa-triggered cycles. A recent report evaluated the effect of different treatment protocols combined with different triggering agents and found that embryos cleaved faster, especially in the early developmental steps, following an antagonist protocol plus GnRHa triggering compared with a GnRHa protocol plus hCG triggering [10]. Our results were similar. However, the stimulation protocols also differed in the aforementioned study groups. Therefore, both stimulation and triggering factors affected the results. In comparison, in our study, both groups followed a GnRH antagonist protocol. Therefore, their result might also be related to triggering factors, rather than the stimulation protocol.

In this study, we found that oocytes were fertilized earlier and that the PNF, t_2_, t_3_, t_5_, and t_6_ intervals were shorter, while we did not detect any difference in the later events. What is the role of early developmental kinetics in subsequent embryo development? Previous studies have compared early and late cleaving embryos and found that significantly more early cleaving embryos were good-quality embryos and the transfer of early cleavage embryos resulted in higher implantation and pregnancy rates [15–17]. Recently, Lemmen et al. reported that the disappearance of pronuclei and first division occur earlier in embryos that implant and cell number is higher on day 2 of embryonic development [18]. Wong et al. found a correlation between reaching the blastocyst stage and the first, second, and third cell divisions [19]. Meseguer et al. evaluated the use of morphokinetics as a predictor of embryo implantation and reported six discriminative morphokinetic parameters (t_2_, t_3_, t_4_, t_5_, cc_2_, and s_2_) that were correlated with implantation [11]. In our study, the durations of t_2_, t_3_, and t_5_ of GnRHa-triggered embryos were shorter than for hCG-triggered embryos. Unfortunately, we could not evaluate the relationship between these differences in early developmental kinetics and pregnancy rates because we did not use embryo developmental kinetics for embryo selection. However, the proportion of optimal embryos based on the relevant morphokinetic variables (s2 and cc2) which are considered to have a strong predictive potential of embryo competence were significantly higher in GnRH agonist triggered group. Thus the optimal time intervals stated above are related to higher implantation potential [17–19].

What causes the difference in the early embryologic developmental kinetics between GnRHa- and hCG-triggered cycles? One explanation might be the oocyte maturation process. Some recent studies focused on the oocyte maturation rate after hCG and GnRHa triggering, and found that the number of oocytes retrieved, percentage of mature oocytes, and number of top-quality embryos were either comparable or in favor of the GnRHa trigger [8, 20]. These findings were related to different physiological events that happened after hCG and GnRHa triggering. Therefore, the effects of these agents differ in duration and receptor activation in some instances [20–22]. The duration of the LH surge after hCG triggering is longer than with GnRHa triggering, *i.e*., days versus 24 h, respectively [18]. LH has a greater impact on AKT and extracellular signal regulated kinase (ERK1/2) phosphorylation and is responsible for granulosa cells proliferation, differentiation, and survival, while hCG generates more intracellular cAMP accumulation, which stimulates steroidogenesis [22]. HCG induces elevated follicular fluid progesterone levels, suggesting that there are differences in the oocyte microenvironment just before ovulation compared with the endogenous LH surge [23]. Recent studies also suggested a potential favorable impact of FSH in the process of nuclear maturation by actively promoting the resumption of meiosis [19, 24, 25]. Erb et al. reported a significantly greater yield of high-quality embryos in the GnRHa-triggered group in donor cycles [26]. They suggested that the longer half-life of hCG causes over-luteinization of the recruited follicles, affecting oocyte and embryo quality.

Although GnRHa triggering seems to favor oocyte maturation and embryo development, one major disadvantage of GnRHa-triggered cycles is the luteolytic effect after GnRHa triggering, which may necessitate adding low-dose hCG for luteal support in fresh IVF cycles [8]. A dual or double trigger using both GnRHa and hCG might improve oocyte and embryo quality, while supporting the luteal phase. Lin et al. found that significantly more oocytes were retrieved, with more mature oocytes, and more embryos cryopreserved, with a significant increase in implantation, clinical pregnancy, and live birth rates, as compared with the hCG-triggered group [27]. Decleer et al. reported a greater number of excellent oocytes and cryopreserved embryos after dual triggering, compared with hCG only [28]. A recent paper recommended prolonging the time interval between ovulation triggering with a GnRHa and oocyte pick-up to overcome any existing impairment in granulosa cell function, oocyte meiotic maturation, or cumulus expansion for patients with abnormal follicular maturation [29]. We did not evaluate the effects of a dual trigger on oocyte development. Future studies evaluating the embryo developmental kinetics after a dual trigger will give more information on the subject.

Our findings suggested favorable embryo developmental kinetics after GnRHa triggering. This may be related to the more physiological maturation process in response to GnRHa as previously reported by others [24–26]. Therefore, another patient-friendly option might be GnRHa triggering combined with transfer of these embryos in a natural thaw cycle with a natural endometrium, especially for patients at high risk of OHSS. Elective vitrification is an alternative embryo transfer strategy to achieve better perinatal outcomes following assisted reproduction technology treatment [30]. Future randomized controlled studies will provide more information.

## Conclusions

In conclusion, the method used to trigger oocyte maturation seems to affect the dynamic parameters of early oocyte and embryo development. Larger randomized controlled studies are needed to evaluate the clinical effects of these findings.
